# The Utility of Diffusion and Perfusion Magnetic Resonance Imaging in Target Delineation of High-Grade Gliomas

**DOI:** 10.1155/2020/8718097

**Published:** 2020-08-10

**Authors:** Qian Fei, Lu-Xi Qian, Yu-Jie Zhang, Wen-Jie Guo, Xiu-Hua Bian, Li Yin, Peng-Wei Yan, Ting-Ting Wang, Pu-Dong Qian, Zhen Guo, Xia He

**Affiliations:** ^1^Department of Radiation Oncology, The Affiliated Cancer Hospital of Nanjing Medical University & Jiangsu Cancer Hospital & Jiangsu Institute of Cancer Research, 42 Baiziting Road, Nanjing, Jiangsu Province, China; ^2^Department of Radiology, The Affiliated Cancer Hospital of Nanjing Medical University & Jiangsu Cancer Hospital & Jiangsu Institute of Cancer Research, 42 Baiziting Road, Nanjing, Jiangsu Province, China

## Abstract

**Background:**

The tumor volume of high-grade glioma (HGG) after surgery is usually determined by contrast-enhanced MRI (CE-MRI), but the clinical target volume remains controversial. Functional magnetic resonance imaging (multimodality MRI) techniques such as magnetic resonance perfusion-weighted imaging (PWI) and diffusion-tensor imaging (DTI) can make up for CE-MRI. This study explored the survival outcomes and failure patterns of patients with HGG by comparing the combination of multimodality MRI and CE-MRI imaging with CE-MRI alone.

**Methods:**

102 patients with postoperative HGG between 2012 and 2016 were included. 50 were delineated based on multimodality MRI (PWI, DTI) and CE-MRI (enhanced T1), and the other 52 were delineated based on CE-MRI as control.

**Results:**

The median survival benefit was 6 months. The 2-year overall survival, progression-free survival, and local–regional control rates were 48% vs. 25%, 42% vs. 13.46%, and 40% vs. 13.46% for the multimodality MRI and CE-MRI cohorts, respectively. The two cohorts had similar rates of disease progression and recurrence but different proportions of failure patterns. The univariate analysis shows that characteristics of patients such as combined with epilepsy, the dose of radiotherapy, the selection of MRI were significant influence factors for 2-year overall survival. However, in multivariate analyses, only the selection of MRI was an independent significant predictor of overall survival.

**Conclusions:**

This study was the first to explore the clinical value of multimodality MRI in the delineation of radiotherapy target volume for HGG. The conclusions of the study have positive reference significance to the combination of multimodality MRI and CE-MRI in guiding the delineation of the radiotherapy target area for HGG patients.

## 1. Introduction

Gliomas are the most common diseases in primary intracranial tumors [1], and high-grade gliomas (HGG), which are also known as malignant gliomas (WHO grades III-IV) [2], account for approximately 85% of primary malignant brain tumors [3]. However, gliomas, especially HGG, have the characteristic of invasive growth [4], making it difficult to perform radical resection [5, 6]. Clinically, surgery combined with postoperative radiochemotherapy is the standard treatment for HGG [7]. A previous study showed that the median survival is 14.6 months [8].

To date, cranial MRI is the most widely used imaging method in the delineation of the radiotherapy target volume with postoperative HGG [9, 10]. However, surgically induced enhancement can be observed by MRI, which is difficult to distinguish from postoperative residual or early tumor recurrence, especially malignant glioma [11]. Studies have confirmed that scattered tumor cells can exist in the peritumoral edema area [12, 13]. In the 2016-2018 NCCN guidelines of central nervous system cancers, the definition of the clinical target volume (CTV) for HGG was not changed, which is gross tumor volume (GTV) plus 1–2 cm margin for grade III and GTV plus 2–2.5 cm margin for grade IV. Given the vague definition of the target volume of the edema area, it is limited that CTV is defined as GTV with a uniform extension in accordance with the NCCN guidelines. Hence, the accurate delineation of the target volume has become the focus of clinical research to improve the efficacy further.

Functional magnetic resonance imaging (multimodality MRI) has developed rapidly in recent years, facilitating the assessment of biological characteristics for the target delineation of postoperative gliomas. Magnetic resonance perfusion-weighted imaging (PWI) can reflect tissue perfusion and microvascular permeability, and infiltrating tumors could be characterized by abnormal permeability [14–16]. It is worth mentioning that malignant glioma principally infiltrates along white matter fiber tracts [17, 18]. Diffusion-tensor imaging (DTI) is a noninvasive MRI technique for assessing the orientation of white matter fibers based on the molecular motion of water in brain tissue [15, 18, 19]. Peritumoral infiltration can be detected by changes in white matter anisotropy and diffusivity [17, 18]. In the clinical treatment of glioma, the application of DTI or PWI can guide the definition of tumor boundary, make the delineation of radiotherapy target area more accurate, realize individualized radiotherapy plans, and save patients from ineffective treatment, thus improving the prognosis of patients. [20, 21]

We hypothesized that the combination of multimodality MRI (PWI, DTI) and frequently used MRI imaging (enhanced T1) could provide a more accurate assessment of tumor infiltration. To confirm the clinical application value of multimodality MRI in guiding the delineation of the radiotherapy target area, we conducted a retrospective analysis to compare the survival outcomes and failure patterns of HGG patients, whose target areas were defined based on the multimodality MRI plus CE-MRI or CE-MRI alone.

## 2. Materials and Methods

### 2.1. Patients

A total of 102 patients with postoperative HGG from May 2012 to May 2016 were retrospectively reviewed in the study. The following are the eligibility criteria for this study: (1) All cases were confirmed by surgical pathology as WHO grade III-IV glioma. (2) The Karnofsky Performance Status (KPS) after surgery was more than 70. (3) All were treated with standard STUPP regimen. (4) All patients had complete follow-up data. Clinicians evaluated whether patients had epilepsy by physical examination and emotional intelligence tests. If the patient had hemiplegia, it was considered a kind of dysfunction. Complete resection refers to the complete resection of the visible tumor. Partial resection refers to the resection of part of the tumor in order to retain some functions, such as language function or motor function. Of the 102 patients, 50 were delineated based on CE-MRI, PWI, and DTI. The other 52 were controlled, and the CE-MRI (enhanced T1) was used to delineate the target areas. Patients from both cohorts came from the same period (2012-2016), and their treatment and examination methods were similar. This retrospective study was approved by institutional review boards (No. 20111025002).

### 2.2. Imaging Acquisition and Processing

All patients were examined with the PHILIPS Archiva 1.5T magnetic resonance imager, Sense NV 16 coil. MRI (conventional scan + enhancement), DTI, and PWI were performed before radiotherapy for postoperative patients. All patients received radiotherapy using a 6 MV X-ray and 3D treatment planning system. We used a plastic face mold for head fixation, laser light for field placement, and then CT continuous enhanced scanning, with a scanning layer thickness of 3 mm to obtain image data, and then imported the images into the planning system. After the CT image was fused with the MR image, the target area was delineated in the corresponding MR image.

The conventional T1WI and T2WI were performed first, axial position T1WI (TR 450 ms, TE 15 ms, FOV 230 × 180 mm, and voxel size 0.9 × 1.14 mm), axial position T2WI (TR 1400 ms, TE 70 ms) with spin echo sequence. DTI examination was performed prior to the injection of the contrast agent. DTI scan uses a single excitation plane echo sequence and 15 diffusion gradient directions under the following parameters: TR, 8087 ms; TE, 75 ms; matrix, 92 × 110; *b* value selected, 0 and 800 s/mm^2^; voxel size, 2 mm; slice thickness, 2 mm; slice gap, 0 mm; and DTI scan duration, 4 min 26 s. Then, the Gd-DTPA contrast agent was injected through the radial vein at a dose of 0.1 mmol/kg and a flow rate of 3 mL/s, and PWI scan, T1WI-enhanced scan was performed. PWI scan uses T2∗-weighted under the following parameters: TR/TE, 1829 ms/40 ms; matrix, 88 × 87; FOV, 224 × 224 mm; voxel size 2.5 × 2.5 mm; flip angle, 75°; slice thickness, 5 mm; slice gap, 0 mm; PWI scan, 40 times; and PWI scan duration, 1 min 20 s.

The PWI raw data were imported into the PHILIPS MR Systems Achieva Release 3.2.3.5′ image processing workstation using built-in analysis software to automatically generate negative integral (NI, relative cerebral blood volume (rCBV)), mean transit time (MTT), index (defined as NI divided by MTT), time of arrival (T0, contrast agent arrival time), and time to peak (TTP, time till contrast agent bolus reaches peak intensity).

The DTI raw image was transmitted to the PHILIPS MR Systems Achieva Release 3.2.3.5′ image processing workstation, and the fiber bundle length, numbers, and FA value of the region of interest of the lesion area were delineated and obtained by the self-contained software.

### 2.3. Diagnostic Criteria

**Figure 1:**
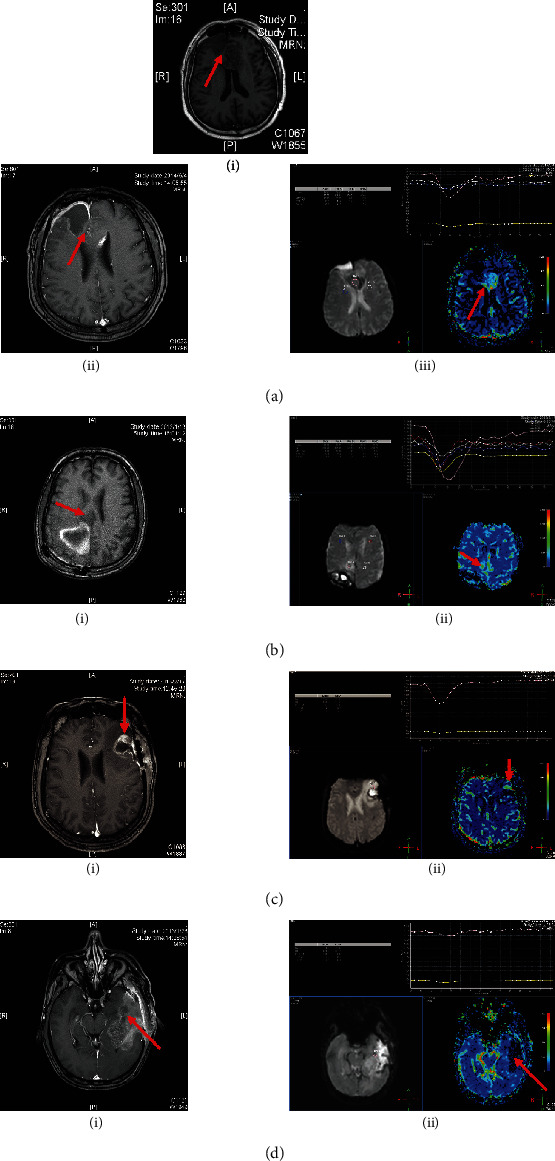
CE-MRI and PWI of the postoperative HGG patient before radiotherapy. (a). CE-MRI and PWI images for a 60-year-old male patient who had double frontal astrocytoma (WHO Grade III). The PWI showed high rCBV of 1.86 in the surgical cavity (iii), while no nonuniform contrast enhancement was found in the same region in the T1 axial image (i) and CE-MRI axial image (ii). (b). CE-MRI and PWI images for a 64-year-old male patient who had the right fronto-parietal glioblastoma (WHO Grade IV). The PWI showed high rCBV of 1.62 in the non-surgical area (ii), but the CE-MRI axial image did not illustrate contrast enhancement (i). (c). CE-MRI and PWI images for a 40-year-old male patient who had left frontal glioma (WHO Grade IV). CE-MRI, nonuniform contrast enhancement in the surgical cavity (i), and PWI shows high rCBV of 2.99 (ii). (d). CE-MRI and PWI images for a 63-year-old male patient who had left parieto-temporal lobe glioblastoma (WHO Grade IV). CE-MRI, nonuniform contrast enhancement in the surgical cavity (i), and PWI shows rCBV is normal (ii).

**Figure 2:**
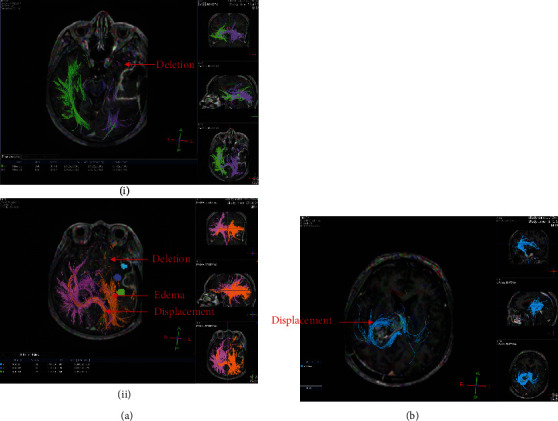
DTI images of the postoperative HGG patient before radiotherapy. (i). DTI images for a 59-year-old male patient who had left temporal lobe glioma (WHO Grade III). Fiber tract disruption and absence outside the surgical cavity. (ii). DTI images for a 64-year-old female patient who had right thalamic glioma (WHO Grade III). The fiber tract was continuous and complete, considering that tumor invasion is not obvious.

MR-enhanced scans that showed the following conditions were considered residual lesions: homogeneity enhancement, linear enhancement, micronodular enhancement, confused and disorderly enhancement, and contrast enhancement within the surgical cavity [22]. For PWI, we selected rCBV = 1.47 as the threshold for progression in this study (Figure 1) [23]. For HGG patients, white matter tract involvement has traditionally been classified as edema, infiltration, displacement, or disruption [24–26], which can be illustrated by DTI (Figure 2). To define whether the fiber tract is involved, we classified the fiber tract disruption and/or absence beyond the surgical cavity as HGG involvement. One or more contrast enhancement nodules in the ependyma and choroid indicate an abnormally thickened contrast enhancement, such as pigtail nodular or enhanced nodular within the spinal cavity.

Failure types include in-field failure and out-field failure. The recurrence of tumor within 50 Gy isodose line is considered an in-field failure. The marginal failure and distant metastasis belong to the out-field failure. The distance between the recurrence center and 50 Gy isodose line is less than 1.5 cm, which is considered a marginal failure. On the contrary, the distance is more than 1.5 cm, which is considered distant metastasis [27, 28].

### 2.4. Target Delineation

**Figure 3:**
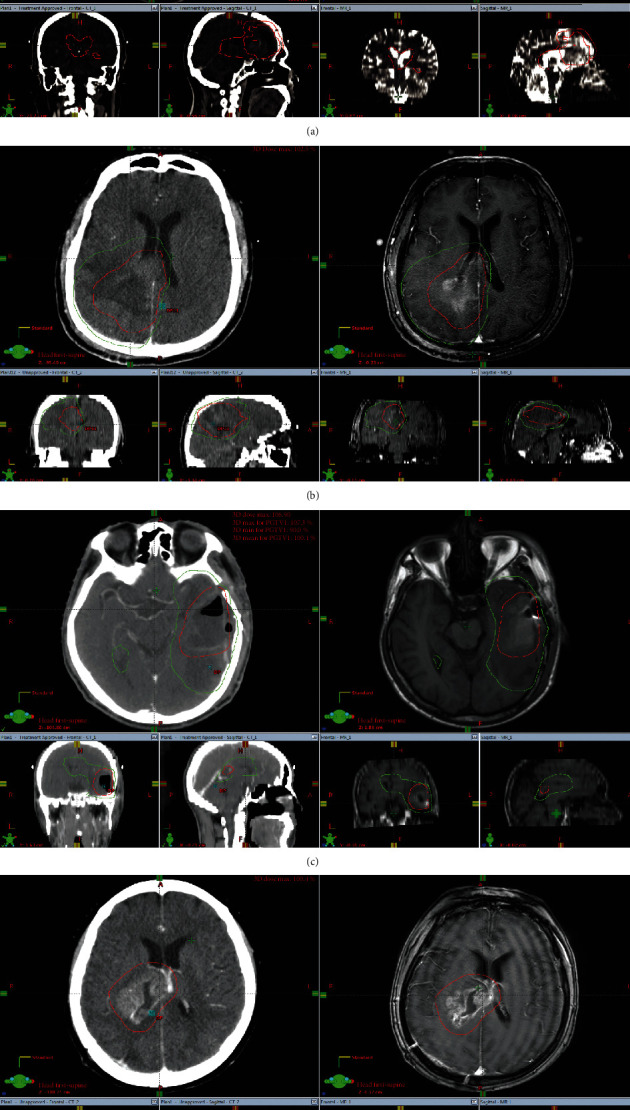
Target volumes of the postoperative HGG patient before radiotherapy. (a). The same patient as Figure 1(a). GTV was resection cavity and high perfusion region; CTV1 covered the GTV + 5 mm margin and received 60 Gy/30 fx; CTV2 covered the CTV1 + 5 mm margin, and the ependyma involved. (b). The same patient as Figure 1(b). GTV was resection cavity and high perfusion region; CTV1 covered the GTV + 5 mm margin and received 60 Gy/30 fx. (c). The same patient as Figure 2(a). GTV was fiber tracts occurring with disruption and/or absence region; CTV1 covered the GTV + 5 mm margin and received 60 Gy/30 fx. (d). The same patient as Figure 2(b). GTV was resection cavity; CTV1 covered the GTV + 5 mm margin and received 60 Gy/30 fx.

The Varian treatment planning system was used to complete target volume delineation. For the patients in the control cohort, the GTV was defined in accordance with the NCCN guidelines by two experienced radiologists (twenty years of experience), using only the axial T1-weighted contrasting images. The GTV was expanded 1–2 cm (CTV) for grade III and up to 2–3 cm (CTV) for grade IV. For the patients in the study cohort, the PWI, DTI, and CE-MRI (enhanced T1) were reviewed, and the target volumes, including areas of the suspicious tumor on PWI/DTI images not obvious on CE-MRI, were defined accurately by two experienced radiologists (twenty years of experience) (Figure 3). In terms of target delineation, we contoured target volume on the functional images, then copied it to the CT-sim images and modified it according to the anatomical images. The definition of CTV1 was resection cavity, high-perfusion region, and fiber tracts occurring with disruption and/or absence with an additional 5 mm margin. CTV2 covered the CTV1 + 5 mm margin, and the area of fiber tracts occurred with sparsity and displacement and the ependyma if any part of it was involved. The target delineation used in this study is similar to the cone-down boost technique. Radiotherapy and chemotherapy were performed in accordance with the STUPP regimen. All patients were treated with the standard STUPP regimen. Considering the vague definition of the target volume of the edema area in the NCCN guidelines, we defined CTV2 based on the DTI and the involvement of cerebral ventricles regardless of the existence of edema.

### 2.5. Statistical Analysis

We used the chi-square or Fisher exact tests to compare categorical data. Survival analysis was performed by using the Kaplan–Meier method, and the log-rank test was used to assess the difference between the two groups. The prognostic factors on overall survival were evaluated by univariate and multivariate analyses. The variables with *P* < 0.3 in the univariate analysis were included in multivariate analyses by using the forward conditional Cox proportional hazards model. Statistical significance was considered at *P* < 0.05. Data were analyzed using SPSS version 22 (SPSS Inc, Chicago, IL).

## 3. Results

### 3.1. Clinical Features

The characteristics of the 102 patients are listed in Table 1. The median age was 50 years (range, 7–75). The ratio between males and females was 11 : 6. Two-fifths of the patients (36%) had anaplastic astrocytoma or oligoastrocytoma (WHO grade III). Epilepsy and mental dysfunction were observed in 16% and 18% of the patients, respectively. All patients received standard STUPP regimen. The median dose of postoperative radiotherapy was 60 Gy (range, 45–72 Gy). Most HGG patients (91 (89%)) received at least 60 Gy, and 11 (11%) received less than 60 Gy. The median interval between surgery and radiotherapy was 4 weeks (range, 2–8).

### 3.2. Survival and Progression

To evaluate the utility of multimodality MRI (PWI and DTI) in the patients with HGG, we compared the survival outcomes of patients for which multimodality MRI plus CE-MRI or CE-MRI alone helped to define the postoperative radiotherapy target volumes. All clinical characteristics were comparable between the two cohorts (Table 1). At a median follow-up of 20 months, 80 (78%) of the patients had died. The median survival benefit was 6 months, and the median survival was 24 months in the multimodality MRI cohort and 18 months in the CE-MRI cohort.

**Figure 4:**
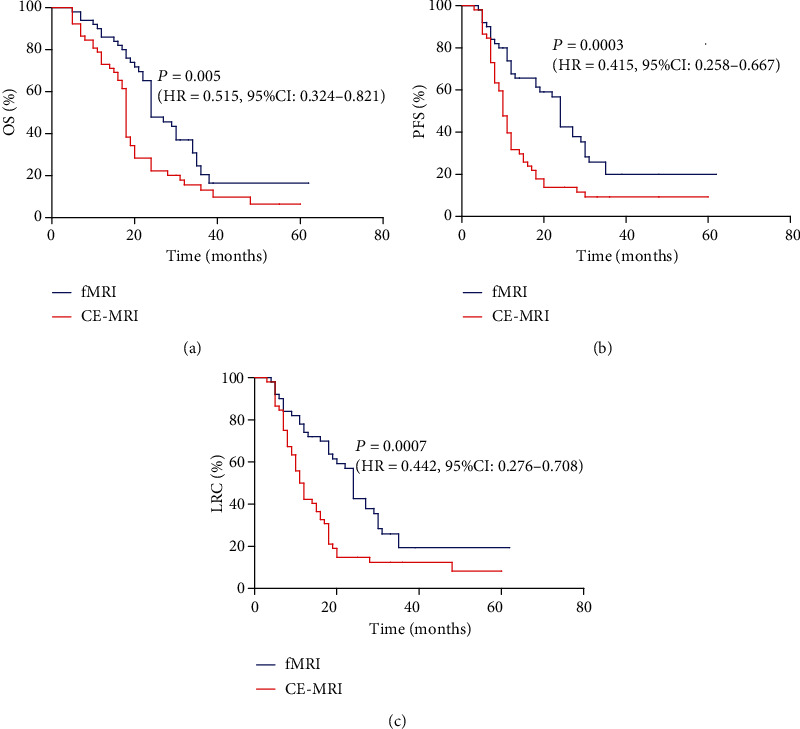
OS, PFS, and LRC for the multimodality MRI and CE-MRI cohorts. (a) OS, (b) PFS, and (c) LRC stratified by multimodality MRI and CE-MRI in 102 patients with HGG.

The 2-year overall survival (OS), progression-free survival (PFS), and local–regional control (LRC) rates were 48%, 42%, and 40% for the multimodality MRI cohort, whereas those for the CE-MRI cohort were 25% (*P* = 0.005, Figure 4(a)), 13.46% (*P* = 0.0003, Figure 4(b)), and 13.46% (*P* = 0.0007, Figure 4(c)), respectively.

### 3.3. Failure Patterns for Multimodality MRI Cohort vs. CE-MRI Cohort

We further analyzed the failure patterns for the patients with HGG in the multimodality MRI and CE-MRI cohorts. Disease progression or recurrence occurred in 64 (62.7%) of the 102 patients. Patients in the multimodality MRI and CE-MRI cohorts had similar rates of disease progression and recurrence, i.e., 62% and 63.5% (*P* = 0.879), respectively. However, the proportion of failure patterns in the two cohorts was different. In the multimodality MRI cohort, 28 (90.3%) of the patients experienced recurrence within the irradiated field (range, 50–60 Gy), and 4 (9.7%) experienced out-field failure (2 cases of marginal failure, 1 case of pituitary metastasis, and 1 case of cerebrospinal fluid metastasis). In the CE-MRI cohort, 26 (78.8%) of the patients experienced recurrence within the irradiated field (range, 50–60 Gy), and 7 (21.2%) experienced out-field failure (2 cases of marginal failure, 2 cases of corpus callosum metastasis, 1 case of pineal gland metastasis, 1 case of contralateral brain metastasis, and 1 case of distant intraparenchymal metastasis).

### 3.4. Univariate and Multivariate Analyses

The results of univariate and multivariate analyses for OS are shown in Table 2. In the univariate analysis, characteristics of patients such as combined with epilepsy (yes or no), the dose of radiotherapy (≥60 Gy or <60 Gy), the selection of MRI (multimodality MRI+CE-MRI or CE-MRI alone) were significant influence factors for OS. However, in multivariate analyses, only the selection of MRI was an independent significant predictor of OS. Thus, 2-year OS was better for patients with multimodality MRI+CE-MRI than for those with CE-MRI alone (HR =1.99, 95% CI: 1.26-3.16, *P* = 0.003).

## 4. Discussion

Previous studies have reported the application of multimodality MRI in gliomas. Some studies indicated that the clinician can capture subtle changes, such as integrity damage and diffusivity changes caused by peritumoral edema or tumor infiltration, in neuronal structures and fibers through multimodality MRI [29–33]. Other studies suggested that multimodality MRI can be used to distinguish high-grade gliomas from metastatic tumors [34–37]. Recent studies have shown that multimodality MRI is a valuable noninvasive tool in differentiating residual/recurrent gliomas from postirradiation cerebral lesion [38, 39]. However, few articles reported the potential value of multimodality MRI in guiding the target delineation for patients with HGG, especially in survival and failure mode analyses. Early in the last decade, Price et al. thought that multimodality MRI could improve the delineation of the radiotherapy target volume for malignant gliomas and potentially guide treatment for tumor infiltration [40]. Recently, Jensen et al. have conducted DTI-driven growth models, which lead to a considerable increase in the Hausdorff distance and reduction in the overlap between the standard and model-derived volume [41]. However, these studies did not report whether multimodality MRI used on target delineation is beneficial for HGG patients themselves and simply focused on the changes in the relevant parameters of target volumes. This study is the first to describe the clinical value of multimodality MRI in the delineation of the radiotherapy target volume for HGG.

In this study, the 2-year OS, PFS, and LRC rates of the patients with HGG in the multimodality MRI cohort were significantly better than those of the patients in the CE-MRI cohort. This result indicated that the survival outcomes of the patients with HGG can improve with the application of multimodality MRI in target delineation. In addition, the 2-year survival rate of the patients in the multimodality MRI cohort was superior to that of the patients in the 2005 historical cohort (48% vs. 26.5%) [8]. However, other confounding factors possibly influenced the results. In the present study, only 64% of the patients were diagnosed with WHO grade IV; however, all patients included in the 2005 historical cohort were histologically confirmed to have glioblastoma (WHO grade IV), a type of high-degree malignant and high-invasive astrocytoma [8]. Meanwhile, the patients in the historical cohort received three-dimensional conformal radiotherapy; in this study, all patients received intensity-modulated radiotherapy [8].

Several studies investigated the failure patterns of the patients with HGG who received postoperative radiotherapy. Chang et al. suggested that 90% of the patients with glioblastoma multiforme failed in the central and in-field localization in both radiotherapy plans (46 Gy for the Radiation Therapy Oncology Group (RTOG) and 50 Gy for M. D. Anderson Cancer Center (MDACC)) [42]. Our results showed that 90.3% of the 31 patients with disease progression and recurrence experienced recurrence within the irradiated field, which is in line with the previous study. Up to now, the majority of studies on failure patterns in glioblastoma showed that in-field failures are the most common form of failure, and marginal and out-field failures are relatively uncommon [43–45]. Furthermore, the different proportions of failure patterns in the two cohorts indicated that the combination of multimodality MRI and CE-MRI to delineate the target volume might reduce the risk of distant metastasis.

Several inherent limitations are related to this retrospective study. First, this study involved glioma patients who were limited to WHO grade III and IV, and the impact of multimodality MRI used for postoperative radiotherapy contouring on the outcomes of low-grade glioma patients remains unknown. Second, the number of HGG patients included in this study and the follow-up time are limited. Thus, further evaluation and a longer follow-up period are needed to confirm the value of multimodality MRI for HGG patients in future studies. Third, in addition to the different reference images of tumor contouring, several other inevitable and objective heterogeneity factors exist between the two cohorts, which are associated with its retrospective nature. Fourth, functional MRI contains many types of magnetic resonance techniques. Only PWI and DTI were evaluated in the research. Basically, the evaluation of PWI and DTI images is subjective, and a unified criterion for assessing tumor is lacking. Radiation therapists and radiologists have to work collectively to reduce errors during the target delineation. Finally, a diagnostic criterion of HGG pseudoprogression remains to be established to date. In the present study, we evaluated the HGG pseudoprogression on the basis of perfusion examination (rCBV) and follow-up data. In sum, large-scale and scientific and prospective studies should be conducted to clarify the utility of multimodality MRI for target delineation in HGG.

## 5. Conclusions

In conclusion, this study suggested that the combination of multimodality MRI and CE-MRI in the progression of target delineation helped improve the survival outcome of patients with high-grade glioma. The application of multimodality MRI can reduce the risk of distant metastasis for patients with high-grade glioma.

## Figures and Tables

**Table 1:** Clinical characteristics. Comparison of the baseline characteristics of the patients for which multimodality MRI or CE-MRI alone helped to define the postoperative radiotherapy target volumes.

Characteristic	All patients (*n* = 102) no. (%)	Multimodality MRI (*n* = 50) no. (%)	CE-MRI (*n* = 52) no. (%)	*P* value
Age (years)				0.92
Median	50	51	49.5	
Range	7-75	7-75	8-72	
Age				0.99
≤50	49 (48)	24 (48)	25 (48)	
>50	53 (52)	26 (52)	27 (52)	
Gender				0.88
Male	66 (65)	32 (64)	34 (65)	
Female	36 (35)	18 (36)	18 (35)	
WHO grade				0.38
III	37 (36)	16 (32)	21 (40)	
IV	65 (64)	34 (68)	31 (60)	
Combined with epilepsy				0.53
Yes	16 (16)	9 (18)	7 (13)	
No	86 (84)	41 (82)	45 (87)	
Mental functions				0.93
Normal	84 (82)	41 (82)	43 (83)	
Dysfunction	18 (18)	9 (18)	9 (17)	
Extent of surgery				0.51
Complete resection	40 (39)	18 (36)	22 (42)	
Partial resection	62 (61)	32 (64)	30 (58)	
Interval between surgery and radiotherapy (wk)				0.96
Median	4	4	4	
Range	2-8	3-8	2-8	
Radiotherapy				
Dose (Gy)				0.98
Median	60	60	60	
Range	45-72	46-72	45-70	
Dose				0.37
≥60Gy	91 (89)	46 (92)	45 (87)	
<60Gy	11 (11)	4 (8)	7 (13)	
Fractions				0.71
Median	30	30	30	
Range	17-48	17-48	20-45	
Concomitant temozolomide	102 (100)	50	52	
Adjuvant-therapy				0.49
Adjuvant temozolomide	23 (23)	12 (24)	11 (21)	
Targeted therapy	17 (17)	7 (14)	10 (19)	

**Table 2:** Univariate and multivariate analyses. Clinical parameters affecting the overall survival of all patients in univariate and multivariate analyses.

Characteristic	Univariable analysis		Multivariable analysis	
	HR (95% CI)	*P* value	HR (95% CI)	*P* value
Age				
>50	1 (referent)			
≤50	0.98 (0.63-1.50)	0.90		
Gender				
Male	1 (referent)			
Female	1.24 (0.79-1.94)	0.34		
Adjuvant-therapy				
Yes	1 (referent)			
No	0.94 (0.60-1.48)	0.80		
Combined with epilepsy				
Yes	1 (referent)		1 (referent)	
No	0.67 (0.37-1.20)	0.18	0.57 (0.31-1.05)	0.07
Mental functions				
Normal	1.21 (0.67-2.19)			
Dysfunction	1 (referent)	0.53		
Dose				
≥60 Gy	0.67 (0.33-1.34)		0.74 (0.36-1.50)	
<60 Gy	1 (referent)	0.26	1 (referent)	0.40
WHO grade				
III	1 (referent)			
IV	0.83 (0.53-1.29)	0.40		
MRI				
Multimodality MRI+CE-MRI	1 (referent)		1 (referent)	
CE-MRI alone	1.81 (1.16-2.82)	0.01	1.99 (1.26-3.16)	0.003

## Data Availability

The data used to support the findings of this study are included within the article.
